# Influence of Cannabinoid Treatment on Trajectories of Patient-Related Outcomes in Chronic Pain: Pain Intensity, Emotional Distress, Tolerability and Physical Disability

**DOI:** 10.3390/brainsci13040680

**Published:** 2023-04-19

**Authors:** Anna Marie Balestra, Katharina Chalk, Claudia Denke, Nashwan Mohammed, Thomas Fritzsche, Sascha Tafelski

**Affiliations:** Department of Anesthesiology and Intensive Care Medicine, Campus Charité Mitte und Campus Virchow Klinikum, Charité—Universitätsmedizin Berlin, Corporate Member of Freie Universität Berlin and Humboldt-Universität zu Berlin, 13353 Berlin, Germany

**Keywords:** chronic pain, cannabinoids, patient-related outcome measures

## Abstract

The treatment of chronic pain with cannabinoids is becoming more widespread and popular among patients. However, studies show that only a few patients experience any benefit from this treatment. It also remains unclear which domains are affected by cannabinoid treatment. Therefore, the present study is novel in that it explores the effects of cannabinoid treatment on four patient-related outcome measures (PROMs), and includes patients with chronic refractory pain conditions who have been given the option of cannabinoid treatment. A retrospective design was used to evaluate the impact of cannabinoid treatment on patients with refractory pain in two German outpatient pain clinics. The present study shows that pain intensity (mean relative reduction (−14.9 ± 22.6%), emotional distress (−9.2 ± 43.5%), pain-associated disability (−7.0 ± 46.5%) and tolerability of pain (−11 ± 23.4%)) improved with cannabinoid treatment. Interestingly, the trajectories of the PROMs seemed to differ between patients, with only 30% of patients responding with respect to pain intensity, but showing improvements in other PROMs. Although the mean treatment effects remained limited, the cumulative magnitude of change in all dimensions may affect patients’ quality of life. In summary, a singular evaluation with pain intensity as the sole outcome does not cover the multidimensional effects of cannabinoids. Therefore, the treatment effects of cannabinoids should be evaluated with different PROMs.

## 1. Introduction

In 2017, medical cannabinoids were introduced into the armamentarium of pain treatment in Germany, despite regulatory institutions not approving any of the available substances with this indication [1]. Growing evidence from meta-analyses shows that cannabinoids used to treat chronic noncancer pain (CNCP) reduce the pain intensity only slightly with a number needed to treat (NNT) of 14–24 patients [2] and a number needed to harm (NNH), for any adverse event, of six. Therefore, treatment with cannabinoids remains controversial, as the risk–benefit ratio does not seem to be in favor of treating pain. In contrast, rising patient interest in cannabinoid treatment can be attributed to the impressive and meaningful symptom improvements that have been reported in the media. Here, cannabinoids are used for the treatment of diverse symptoms, but they also affect quality of life in chronic pain patients, such as improving impairment in their daily activities, anxiety or emotional distress and even tolerability of pain. Obviously, significant discrepancies exist in patient reports and outcome measurements of pain in clinical trials. The traditional outcomes reported in RCTs, such as the numeric rating scale for pain, do not represent optimal patient-related experiences of change in illness. As described recently, chronic pain patients react with significant heterogeneity to strategies against changes such as external stressors, and may require different mental, physical or social coping activities [3]. Cannabinoid studies often report average pain intensity and pain relief, and some report emotional and physical functioning and side effects. Importantly, patient perspectives on pain management may differ from physicians’ perspectives [4]. The VAPAIN (validation and application of patient-reported outcome domains to assess multimodal pain therapy) [5] and IMMPACT (Initiative on Methods, Measurement and Chronic Pain Assessment in Clinical Trials) [6] trials revealed that harmonizing outcome measures in clinical trials will require adaptation to specific diseases and settings with patient perspectives to be included. In summary, the response to cannabinoid treatment in an outpatient setting requires specific patient-related outcome measurements (PROMs) that are currently not available. Based on previous experiences of using cannabinoids in chronic pain, we hypothesized that the trajectories of different PROMs may differ in individual patients undergoing cannabinoid treatment. Therefore, we evaluated outcome measurements addressed during treatment with medical cannabinoids from the perspectives of patients, and assessed related effects in the outpatient care of chronic pain. 

## 2. Materials and Methods

### 2.1. Study Design and Inclusion Criteria

The present retrospective observational study included patients undergoing long-term treatment in two university-affiliated specialized outpatient pain clinics in Berlin, Germany. The departments are located at the Charité Campus Mitte and Charité Virchow Klinik. Both clinics focus on outpatient care and provide a wide spectrum of pain interventions, including pharmacological, psychological and physical therapy, thereby composing an ambulatory, multimodal, interdisciplinary therapeutic strategy.

Cannabinoid treatment for pain conditions is currently available for patients with severe disease and refractory pain that is not alleviated by standard of care measures. Following a formal request for the assumption of cost from the statutory health insurance of patients, this therapeutic option can be provided.

There are various cannabinoid-based therapeutics on the market that differ in terms of their active ingredient content and evidence [1]. Based on our initial scientific findings and clinical experience, medication was selected depending on the patient’s chronic pain disorder, accompanying symptoms and side effects. Recent reviews differ in their potential cannabinoid indications and dosing regimens, and there remains significant uncertainty regarding the choice of optimal compounds [7,8]. Therefore, the selection of cannabinoids prescribed by the pain department was made through team consensus.

The focus of this study is patients with chronic pain conditions lasting at least six months. In this population, different pharmacologic and nonpharmacologic interventions were evaluated and failed to sufficiently alleviate pain. All patients received pretreatment based on a bio-psycho-social model of disease and were, therefore, seen by physicians and pain psychologists in our pain clinic. Ambulatory multimodal therapy comprised pharmacological, psychological and physiotherapeutic interventions.

For the purpose of this study, we included adult patients with chronic refractory pain conditions who had been given the option of cannabinoid therapy from 2017 to 2020. All patients included in this study were adults suffering from long-lasting chronic pain. Patients were excluded if their data were incomplete or if cannabinoid therapy had been provided elsewhere. 

### 2.2. Data Assessment and Measurement

For the purpose of this study, all available routine data were evaluated from patients’ medical records. During every patient visit to the outpatient pain clinic, we assessed patients using a standardized questionnaire based on a validated German pain questionnaire created by the German Pain Society (Deutsche Schmerzgesellschaft e.V. as a part of the European Federation of IASP) [9]. The questionnaire was established in 1998 for use in specialized pain facilities, and was designed to evaluate the therapy process. The questionnaire assesses important aspects of the pain experience from the chronic pain patient’s perspective. For the purpose of this study, we especially focused on the following PROMs: 

#### 2.2.1. PROM: Pain Intensity

To assess the overall intensity of pain in the main pain area during the last week, patients were asked “How would you rate the average severity of your main pain in the past week?”. This measurement was reported on a numeric pain scale (NRS), with 0 representing no pain at all and 10 representing maximum pain intensity. 

#### 2.2.2. PROM: Emotional Distress

Emotional distress is strongly associated with pain and is, therefore, an important aspect of treatment. Patients answered the following question on a 0–10-point Likert scale: “How would you rate your emotional distress in the past week?” A score of 0 represented no emotional distress, and 10 represented maximum emotional distress. 

#### 2.2.3. PROM: Tolerability of Pain

The tolerability of pain is the extent to which the intensity of pain influences one’s own pain-related tolerance level [10]. Patients reported their tolerability of pain using a Likert scale for the question “How would you rate the tolerability of pain during the past week?”. The scores were interpreted as follows:

1. “Not relevant, I have no pain at all”;
2. “I can tolerate may pain well”;
3. “I can hardly tolerate my pain”;
4. “I am unable to tolerate my pain any longer”.

#### 2.2.4. PROM: Pain-Associated Disability

The subjective experience of pain-associated disability has a substantial impact on the development of chronic pain [11]. Patients answered the following question on a 0–10-point Likert scale: “How would you rate interference of pain with daily activities (work, household, social life)?”. A score of 0 represented no interference, and 10 represented maximum “inability to perform any activities”.

### 2.3. Statistical Analysis

This descriptive report uses means and standard deviations for discrete data, medians and 25–75% quartiles for ordinal data or data with skewed distribution, and numbers with percentiles for dichotomous data. Statistical significance was determined using Mann–Whitney-U tests or Fisher’s exact tests. A multivariate logistic regression model was created for each of the four PROMs, including the potentially confounding factors on the independent variable (response to PROMs). To define response, a minimal relevant difference of a 30% reduction in pain-associated symptoms in the PROM domain was defined and calculated as the relative difference from the baseline to the follow-up visit after at least 6-months of therapy. PROMs were, therefore, converted to a binary response variable as an independent variable in the multivariate regression model. Based on previous studies, and to allow for comparability between the different PROMs, the following cofactors were included in all the models: patient age, female sex, cancer pain, neuropathic pain, psychologic comorbidity, duration of chronic pain therapy in months, concomitant co-analgesic use, concomitant opioid use and cannabinoid type (THC versus THC/CBD compound). The regression model fit was assessed using Hosmer–Lemeshow tests, as well as comparative backward-selection regression models. For the purpose of this observational study, data analyses were performed using SPSS 29 (IBM), with *p* < 0.05 indicating statistical significance. Alpha-level adaptation was not applied, according to multiple testing, due to the exploratory study design.

## 3. Results

**Figure 1:**
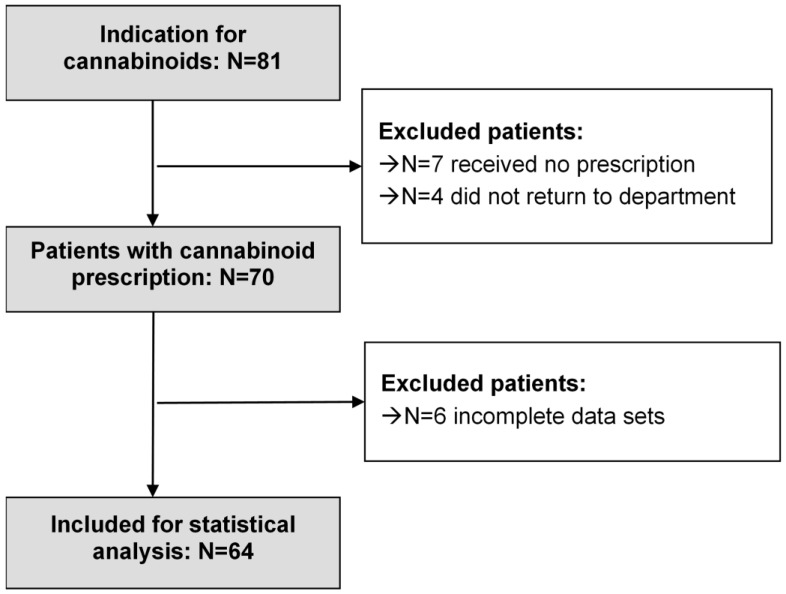
Flow diagram of the inclusion criteria.

In both outpatient pain clinics, more than ten thousand patient contacts were made over a period of four years. Of this population, N = 81 patients were identified as having undergone new cannabinoid treatment during the study period. These chronic pain patients were unresponsive to previous therapeutic interventions, and therefore, cannabinoid treatment was started following the team’s decision. For each of these patients, a formal request was sent to their health care insurance providers to cover the costs of therapy. Altogether, 65% of patients were identified at Campus Charité Mitte and 35% at Campus Virchow Klinikum. Out of these eighty-one patients, N = 70 patients received a prescription for cannabinoids in our two departments. After assessing the patients during a follow-up, complete data were available for analysis in N = 64 cases in the study population (Figure 1).

### 3.1. Characteristics of Patients

The study population comprised patients with various pain conditions that had lasted for more than 6 months (Table 1). The study population consisted of 47% women and showed a wide range of ages: 12.5% of patients were younger than 40 years, and 30% of patients were older than 70 years. A total of 52% of the sample (N = 33) comprised patients with a diagnosis of chronic pain disorder with somatic and psychological factors, which was diagnosed according to the ICD-10. This involves a chronic pain disorder with physiological origins, but whose severity and course are significantly influenced by psychological processes [12].

Pain due to malignancy was found in 29.7% of patients (N = 19), and 28.1% of patients (N = 18) suffered from chronic neuropathic pain. Treatment with co-analgesics was provided in N = 43 patients (67.2%), and N = 35 patients (54.7%) received opioids. Cannabinoid treatment was provided at the time of the study, with a median total treatment duration of 25 months (25–75% quartiles: 12–44), and with the longest treatment duration documented at 156 months. The PROMs were assessed within a time frame of about twelve months after the initiation of cannabinoid therapy.

### 3.2. Cannabinoid Therapy

Of N = 64 patients, most patients (N = 49, 77%) received dronabinol as a prescribed substance. Other preparations included nabiximols (N = 13, 20%), as well as pharmacological preparations (Tilray^®^ 10/10, N = 1, 2%) and cannabinoid flowers (N = 4, 6%). 

In the course of uptitration, N = 9 patients (14%) discontinued cannabinoid therapy, three with nabiximols and six with dronabinol. The reasons reported by the patients were mainly side effects (N = 2 nausea, N = 1 dizziness, N = 1 progressive heart failure, N = 1 angina pectoris), insufficient efficacy (N = 2) and the wish to receive cannabinoid flowers for inhaling, which was not supported by health care insurance (N = 1). 

### 3.3. Change in the Use of Opioids

Together with the patients, a dose reduction or discontinuation of the use of an ongoing opioid was discussed as one goal of pain therapy via treatment with cannabinoids. The goal of discontinuation was achieved by N = 14 patients (22%), a dose reduction was achieved by N = 6 (9%) and opioid use was maintained in N = 14 (22%) patients.

### 3.4. Reported Effects on Health Status (PROMs)

**Figure 2:**
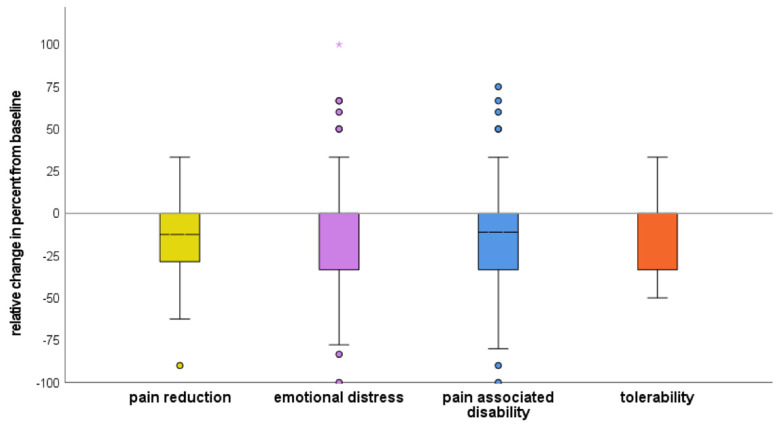
PROM trajectories during treatment with cannabinoids. Colored dots represent data points (outliers) that have 1.5* to 3.0* interquartile range to the 1st or 3rd quartile. Stars indicate data points (outliers) that have a distance greater than 3.0* interquartile range.

The following are the results of the changes in PROMs due to cannabinoid treatment (Table 2, Figure 2).

#### 3.4.1. Pain Intensity

The mean patient-rated pain intensity showed a significant change after patients started treatment with a cannabinoids (*p* < 0.001, Table 2). Patients reported a mean change in mean pain intensity of −1 ± 1.8 points. Based on the criterion of a clinically relevant reduction in pain intensity of at least 30%, N = 14 patients (22%) were defined as responders.

Multivariate logistic regression analysis revealed that patient age and the duration of chronic pain (in months) were predictors of at least a 30% reduction in mean pain intensity under cannabinoids (Table 3).

#### 3.4.2. Pain-Associated Disability

The patients rated their pain-related physical disability on a Likert scale from 0 to 10 before cannabinoid therapy; the mean score was 6.9 ± 2.2. This pain-related physical disability was reduced to 5.8 ± 2.4 points (*p* < 0.001) after the start of treatment. This resulted in a mean change in patients’ disability of 1.1 ± 2.3 points (Table 2). 

Based on the response criterion of a clinically relevant reduction in pain-associated disability of at least 30% in NRS points, N = 20 patients (31.3%) were defined as responders.

Multivariate logistic regression analysis showed that cannabinoid type was significantly associated with at least a 30% reduction in pain-associated disability under cannabinoids (Table 4).

#### 3.4.3. Emotional Distress

In addition to the reduction in pain intensity, improving emotional distress is a relevant goal of cannabinoid treatment. Before cannabinoid therapy, the patients rated their emotional distress on a Likert scale from 0 to 10 (with 0 meaning "good” to 10 meaning “extremely bad”), and the mean score was 5.9 ± 2.5. After treatment, this emotional distress score was reduced to 5.1 ± 2.6 points (*p* = 0.007). Thus, there was a mean change in emotional distress of 0.8± 2.3 points (Table 2).

For emotional distress, N = 19 patients (29.7%) could be defined as responders as they exhibited a clinically relevant change of at least 30% in NRS points.

The multivariate logistic regression analysis for the association between at least a 30% reduction in emotional distress and different variables under cannabinoids revealed no significant predictors (Table 5).

#### 3.4.4. Tolerability of Pain

The patients rated the tolerability of their pain on a Likert scale from 1 to 4 (1 = “I have no pain” to 4 = “I can tolerate it badly”). Before cannabinoid treatment, patients reported poor tolerability of their pain, with a mean of 3.3± 0.7. After the beginning of treatment, this decreased to 2.9 ± 0.8 out of 4 points (*p* < 0.001), resulting in a mean change in tolerability of 0.4 ± 0.8 points (Table 2). For pain tolerability, N = 16 patients (25%) could be defined as responders, on the assumption of a clinically relevant change in this variable of at least 30% in NRS points. Multivariate logistic regression analysis indicated that concomitant opioid use was significantly associated with at least a 30% reduction in pain tolerability under cannabinoids (Table 6).

#### 3.4.5. Overlap of Responses in the Four PROM Trajectories

For the analysis of PROM trajectories (Figure 2), the reported response criteria were used and contrasted in their four categories. There was no significant change in PROMs during cannabinoid treatment for N = 27 patients (42.2%), and there was at least one significant change for N = 37 patients (57.8%). Notably, N = 10 patients (16%) reported more than three responsive trajectories of pain perception.

## 4. Discussion

This study revealed distinct differences in patient-related outcome measurements following cannabinoid therapy in a small number (N = 64 out of 81) of patients with chronic pain that was refractory to previous multimodal treatment. Although the magnitudes were weak, alleviated pain intensity and improved ability to perform daily activities were the most responsive trajectories, with 22% (NNT~5) and 31% (NNT~3) of patients exhibiting at least a 30% improvement in these domains. Approximately 42% of patients were non-responders to cannabinoids in all four PROMs, and only a 5% response rate was observed in all four PROMs. However, the median change in PROMs significantly improved in all four domains, and a relevant frequency of 30% of all patients reduced their dose or ended opioid therapy. 

To assess the intensity of chronic pain, randomized controlled trials were included in a recent meta-analysis of different diseases. Against a placebo, small but significant effects were described for dronabinol and nabiximol, whereas nabilone, compared with the active control, showed no significant effect [13]. Notably, our data fit with those of a recent meta-analysis reporting on the magnitude of the effectiveness of cannabinoids for non-cancer pain [14]. The mean pain intensity was reduced by less than 2 in 10 points, and 21% of patients reported clinically relevant pain relief.

In our population, regarding emotional distress, there was a 30% improvement in the magnitude of symptom severity in 29% of patients. This finding has not been previously evaluated with respect to a specific patient-related outcome measurement, and there is no good evidence for major depression. For anxiety, very few data are available, with just one randomized trial demonstrating a modest short-term effect of cannabidiol [15].

Notably, in our population, 22% of patients were able to end opioid treatment, and approximately 10% reduced their doses. This finding is of interest, especially as our population typically received an oral, low-dose cannabinoid, and is in line with findings reporting an opioid cessation rate of 64% [16] and reporting a more than a 50% opioid dose reduction [17].

In our study, we were not able to precisely monitor patients’ discontinuation of cannabinoids. It would be of interest for further research to identify reasons for cannabinoid discontinuation. In a recent systematic review, psychiatric events were the most common adverse event leading to the discontinuation of cannabinoids [18]. Although it is a known predisposing factor, the pathophysiological mechanism, especially in adolescence, is still under investigation [19]. Further events were cognitive impairment, accidents and injuries, which were estimated to occur in <5% of patients [18]. In our department, patients received medical cannabinoids only after a team discussion incorporating a psychological evaluation and an assessment of the patient’s risk of develop cannabinoid-associated psychiatric disorders. Additionally, cannabinoid treatment should occur following shared decision making with the patient, after they have been informed of the risks and benefits of the chosen compounds. 

Different effects of cannabinoids are closely linked to the interesting intrinsic effects of cannabinoids in mammals. CB1 and CB2 are two G-protein-coupled receptors that can be activated by cannabinoids, and play an important role in the regulation of pain. CB1 receptors are mainly found in the central nervous system, while CB2 receptors are mainly found in the peripheral nervous system and in the cells of the immune system. Moreover, the anti-inflammatory effects of cannabinoids have been linked to the important role of inflammation in different pain disorders. Therefore, both rheumatoid diseases [20] and chronic immune-mediated gastrointestinal diseases have been considered potential indications for cannabinoids [21]. 

More recently, GPR55 was identified in various tissues, including in the brain, immune system and peripheral nervous system [1,22]. 

THC, the most-evaluated active ingredient in cannabis, binds to CB1 and CB2 receptors. At the CB1 receptors in the brain, it triggers more psychoactive effects, such as euphoria, sedation and appetite stimulation. The CB2 receptor, which is also described in cells of the immune system and in the peripheral nervous system, regulate inflammation, pain and other immune reactions [1,22]. 

Interestingly, THC can also bind GPR55 receptors, producing various effects, including the regulation of inflammation, pain and neurological disorders. CBD binds to CB1 and CB2 receptors with a much lower affinity compared with THC. CBD is not a competitive antagonist, but influences the effects of other substances that bind to CB1 and CB2. CBD acts as an inverse agonist and significantly attenuates the effects of other substances at the receptors [1,22]. It can therefore be assumed that cannabinoids not only act on the perception of pain, but can also have effects on tension and emotional distress associated with pain. 

The choice of PROMs for cannabinoid therapy should be adapted to the specific needs of the chronic pain patient. Especially complex experiences such as pain are insufficiently identified by simplified measurements, such as single numeric rating scale data, when suffering is exacerbated by functional limitations, anxiety and depression, an inability to perform daily activities, ineffective coping strategies and a lack of pain tolerability [23]. 

Approximately twenty years ago, core outcome domains for chronic pain trials were recommended, including pain, physical impairment and emotional distress [6]. In more recent data on chronic pain, trials have incorporated such different outcomes to underscore the treatment effects of multimodal interventions [24,25,26]. When pain intensity (NRS) may not reflect the overall treatment effect, differentiating changes in different trajectories of pain is not only a scientific aspect, but also a clinical need. Therapeutic goals are to be defined individually when treatments such as opioids, cannabinoids or invasive pain treatment are planned. From this point of view, emotional distress, pain-associated disability and tolerability of pain may be accepted as aims for the treatment of chronic pain. Moreover, against the background of the broad inherent pharmacological effects described [1], cannabinoids have been assessed for the control of a large variety of diseases or symptoms, such as spasticity, nausea and vomiting, appetite modulation and anorexia nervosa, amyotrophic lateral sclerosis, irritable bowel syndrome, multiple sclerosis, Huntington’s chorea, epilepsy, dystonia, Parkinson’s disease, glaucoma, attention deficit hyperactivity disorder, anxiety, dementia, depression, schizophrenia, PTSD, sleeping disorders, SUD and Tourette’s syndrome [13]. It should be noted that the treatment of these diseases and symptoms may reflect different underlying pathologies and should, therefore, undergo separate assessment; moreover, such diseases may respond to interventions other than cannabinoids. In the case of cannabinoid treatment in these indications, especially in patients in whom different symptoms and diseases are targeted with cannabinoids, perspectives from different specialties should be obtained to define treatment aims and to guide cannabinoid treatment.

## 5. Strengths and Limitations

The present bicentric study is the first evaluation of patients from two outpatient centers who are receiving medical cannabinoids, in the context of the multimodal treatment of chronic pain, that focuses on different trajectories of pain-related symptoms. However, chronic pain populations in university-affiliated centers may differ from those in other settings. Although it is retrospective in nature, the strength of this trial lies in its stringent assessment of the long-term PROMs implemented in routine care. However, other unmeasured outcomes of potential interest were not assessed, and therefore, did not undergo further exploration in this study. For example, although we possessed good data on co-analgesic and opioid use in this cohort, NSAID usage was not documented with sufficient precision for data analysis, as patients received such drugs often over the counter. As an interesting alternative study design, patients’ and physicians’ perspectives could also be evaluated using a qualitative study methodology. Although no causal inferences can be made based on non-randomized trials, our data were consistent with previous prospective clinical trials, and may lead to a better understanding of PROMs in cannabinoid treatment.

## 6. Conclusions

Despite about 60% of patients responding to cannabinoid treatment, there is relevant variability in their responses in different domains of outcome measurements. Regarding future research, there is a need to define endpoints for clinical trials addressing PROMs adjacent to pain intensity.

## Figures and Tables

**Table 1:** Demographic and clinical characteristics of study population.

	N = 64
Age (years), mean ± standard deviation	62.1 ± 16.2
Median [25–75% quartiles]	66 (51–75)
Min–max	22–90
Female sex, N (%)	30 (47%)
Primary indication diagnosis for cannabinoids	
Chronic pain with somatic and psychologic factors	33 (51.6%)
Malignancy	19 (29.7%)
Neuropathic pain	18 (28.1%)
Concomitant pharmacologic treatment	
Co-analgesics (gabapentinoids, antidepressants)	43 (67.2%)
Opioids	35 (54.7%)

**Table 2:** Change in pain intensity, physical disability, pain tolerability and emotional distress before and after starting cannabinoid treatment.

	Before Treatment (M ± SD)	Under Treatment (M ± SD)	Relative Reduction	*p*–Value
Mean pain intensity	6.7 ± 1.8	5.6 ± 2.0	−14.9 ± 22.6%	<0.001 *
Pain-associated disability	6.9 ± 2.2	5.8 ± 2.4	−9.2 ± 43.5%	<0.001 *
Emotional distress	5.9 ± 2.5	5.1 ± 2.6	−7.0 ± 46.5%	0.007 *
Tolerability of pain	3.3 ± 0.7	2.9 ± 0.8	−11 ± 23.4%	<0.001 *

M: mean. SD: standard deviation. * Significant difference between parameters before and under treatment.

**Table 3:** Multivariate logistic regression analysis of the association between at least a 30% reduction in mean pain intensity and cofactors under cannabinoids.

Variables	*p*-Value	Exp (B) Odds Ratio	95% CI for Exp (B)	
			Lower	Upper
Patient age	0.031 *	1.065	1.006	1.127
Female sex	0.407	0.498	0.096	2.585
Cancer pain	0.948	1.059	0.189	5.934
Neuropathic pain	0.238	3.536	0.434	28.796
Psychologic comorbidity	0.185	4.212	0.501	35.385
Duration of chronic pain in months	0.045 *	1.025	1.001	1.051
Concomitant co-analgesic use	0.335	0.442	0.084	2.321
Concomitant opioid use	0.458	1.884	0.354	10.018
Cannabinoid type (THC/CBD compound versus THC)	0.060	5.454	0.934	31.840

Goodness-of-fit was assessed using the Hosmer–Lemeshow test (*p* = 0.690). * *p* < 0.05.

**Table 4:** Multivariate regression analysis of the association between at least a 30% improvement in pain-associated disability and different variables under treatment with cannabinoids.

Variables	*p*-Value	Exp (B) Odds Ratio	95% CI for Exp (B)	
			Lower	Upper
Patient age	0.251	1.024	0.983	1.068
Female sex	0.144	2.989	0.688	12.983
Cancer pain	0.877	1.122	0.262	4.802
Neuropathic pain	0.440	0.568	0.135	2.386
Psychologic comorbidity	0.151	3.834	0.612	24.014
Duration of chronic pain in months	0.217	1.014	0.992	1.035
Concomitant co-analgesic use	0.118	0.318	0.076	1.336
Concomitant opioid use	0.904	1.088	0.278	4.255
Cannabinoid type (THC/CBD compound versus THC)	0.008 **	9.091	1.800	45.916

Goodness-of-fit was assessed using the Hosmer–Lemeshow test (*p* = 0.348). ** *p* < 0.01.

**Table 5:** Multivariate regression analysis of the association between at least a 30% improvement in emotional distress and different variables under cannabinoid treatment.

Variables	*p*-Value	Exp (B) Odds Ratio	95% CI for Exp (B)	
			Lower	Upper
Patient age	0.212	1.028	0.984	1.074
Female sex	0.922	1.068	0.284	4.015
Cancer pain	0.976	1.022	0.252	4.142
Neuropathic pain	0.111	3.778	0.736	19.390
Psychologic comorbidity	0.996	1.003	0.231	4.365
Duration of chronic pain in months	0.207	1.013	0.993	1.033
Concomitant co-analgesic use	0.326	0.512	0.135	1.948
Concomitant opioid use	0.160	2.589	0.687	9.759
Cannabinoid type (THC/CBD compound versus THC)	0.975	0.976	0.208	4.578

Goodness-of-fit was assessed using the Hosmer–Lemeshow test (*p* = 0.348).

**Table 6:** Multivariate regression analysis of the association between at least a 30% improvement in tolerability and different variables with cannabinoid treatment.

Variables	*p*-Value	Exp (B) Odds Ratio	95% CI for Exp (B)	
			Lower	Upper
Patient age	0.733	1.008	0.963	1.055
Female sex	0.835	1.164	0.278	4.873
Cancer pain	0.063	4.410	0.924	21.036
Neuropathic pain	0.935	0.938	0.202	4.357
Psychologic comorbidity	0.368	2.158	0.404	11.516
Duration of chronic pain in months	0.123	1.017	0.996	1.038
Concomitant co-analgesic use	0.134	3.449	0.682	17.438
Concomitant opioid use	0.019 *	6.297	1.347	29.264
Cannabinoid type (THC/CBD compound versus THC)	0.911	1.097	0.215	5.595

Goodness-of-fit was assessed using the Hosmer–Lemeshow test (*p* = 0.039). * *p* < 0.05.

## Data Availability

The data are not publicly available due to [their containing information that could compromise the privacy of research participants].
